# Antiferrodistortive and Ferroeletric Phase Transitions in Freestanding Films of SrTiO_3_

**DOI:** 10.1021/acs.nanolett.4c05664

**Published:** 2025-05-01

**Authors:** Ludmila Leroy, Shih-Wen Huang, Chun-Chien Chiu, Sheng-Zhu Ho, Janine Dössegger, Cinthia Piamonteze, Yi-Chun Chen, Elsa Abreu, Alessandro Bombardi, Jan-Chi Yang, Urs Staub

**Affiliations:** † PSI Center for Photon Science, Paul Scherrer Institute, Forschungsstrasse 111, 5232 Villigen, Switzerland; ‡ Department of Physics, National Cheng Kung University, Tainan 701, Taiwan Center for Quantum Frontiers of Research & Technology (QFort), National Cheng Kung University, Tainan 701401 Taiwan; § Institute for Quantum Electronics, ETH Zürich, Auguste-Piccard-Hof 1, 8093 Zürich, Switzerland; ∥ Diamond Light Source Ltd, Diamond House, Harwell Science & Innovation Campus, Didcot, Oxfordshire OX11 0DE, U.K.

**Keywords:** SrTiO_3_, freestanding film, ferroelectricity, ferroelectric soft mode, antiferrodistortive transition

## Abstract

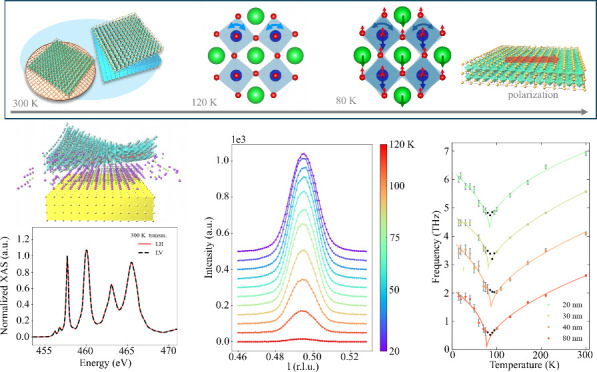

Thin films’ properties can be greatly influenced by their supporting growth substrates. Even in the so-called strain-free heterostructure films, it is still unclear whether there will be no interfacial electronic reconstructions induced by the underlying substrates. Here, we report the studies of SrTiO_3_ (STO) films in the freestanding form (FS) with a thickness ranging from 20 to 80 nm. These STO films, by default, are in a strain-free state; they exhibit distinct properties not seen in both bulk and strain-free heterostructure forms. Our films show an enhanced antiferrodistortive (AFD) phase transition temperature with a preferential in-plane rotation axis for the TiO_6_ octahedra. The anisotropic Ti orbital occupancy around the surface signals the departure of its properties from the bulk. Moreover, we have found that the in-plane ferroelectricity can be strengthened by the reduced dimensionality, establishing that the dimensionality control is an important factor for enhancing STO’s ferroelectric response.

SrTiO_3_ (STO) is an incipient ferroelectric (FE) perovskite.^1−5^ It also displays an antiferrodistortive (AFD) structural phase transition at around 105 K.^6^ This transition, from a high-temperature cubic phase to a low-temperature tetragonal phase, is driven by the rotation of TiO_6_ octahedra and the doubling of the primitive unit cell. For decreasing temperatures, the polar ferroelectric TO1 mode softens and results in a drastic increase in dielectric constant.^5,7^ These phenomena signal the proximity to ferroelectricity.^8−11^ Due to quantum fluctuations that suppress ferroelectricity, STO maintains its paraelectric nature down to the lowest temperature.^5^ STO is one of the most investigated materials in the form of oxide thin films and heterostructures due to its rich response to chemical changes, isotopic substitution, and strain.^12−17^ For example, strained epitaxially grown STO films display ferroelectricity up to room temperature.^18^ Doping a very low concentration of carriers into insulating STO can induce the dilute superconductivity.^16,19,20^ Strained and strain-free STO films grown on supporting substrates, as well as the single crystal STO, are relaxor ferroelectrics.^21^ It has been reported that introducing a buffer layer like SrRuO_3_ (SRO) between the STO substrate and the top STO layer promotes a strain-free STO layer. In such a heterostructure, it has been claimed that polar nanoregions (PNR) with short correlation lengths, presumably precursors of macroscopic ferroelectricity, exist in the top STO layer.^21^ Observation of PNRs indicates that near the surface region of STO, confined in a reduced dimensionality environment, the tendency to stabilize ferroelectricity can be different from that of bulk.

The relationship between AFD and ferroelectricity in the STO is far from simple. Experimental studies show that compressive epitaxial strain stabilizes the out-of-plane FE polarization, while tensile strain favors the in-plane polarization.^18^ Strain suppresses the AFD transition, thereby enhancing ferroelectricity and increasing the FE transition temperature.^11,18^ Spectroscopic studies further reveal that the soft phonon mode frequency, which plays a crucial role of FE transition, is highly strain-dependent.^22,23^ Density functional theory (DFT) studies found that AFD will compete with the rise of ferroelectricity when the TiO_6_ octahedral rotation angles are small, while they cooperate when the angles are large.^24^ Moreover, quantum confinement in STO can shift the balance between AFD and the polar TO1 modes and stabilize one over the other.^25^ However, the DFT studies are yet to be validated because of the limitation on the experimental materials platform: the interfacial interactions can complicate the study of intrinsic properties of the STO top layer as one cannot convincingly exclude the influence from the substrate even in the so-called strain-free heterostructure. Nonetheless, this limitation can be circumvented by using freestanding (FS) films because they do not have growth substrates epitaxially attached to them. Thin FS films also allow experiments to be performed in transmission geometry with soft X-rays, which is advantageous for probing the intrinsic properties of whole materials without being limited to the surface region or influenced by the measurement geometry. As we will show in this report that using X-ray diffraction, X-ray absorption linear dichroism, and THz time domain spectroscopy to study the FS films with different thicknesses, we have revealed their different properties not seen even in the so-called strain-free heterostructure films.

## Film Fabrication and Characterization of the Crystal Structure

**Figure 1:**
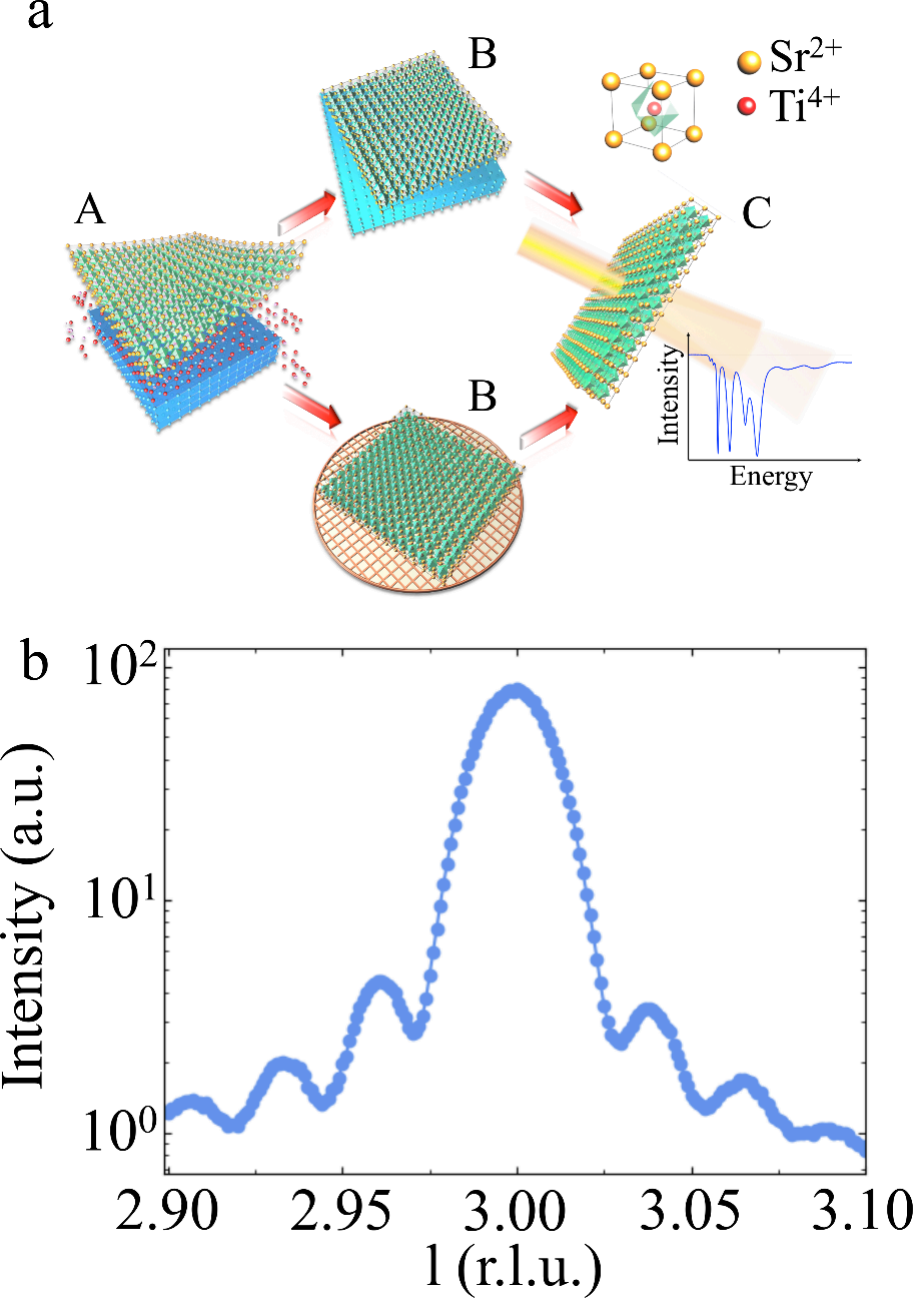
(a) Schematic illustration of the fabrication process of FS films with the following steps: (A) wet etching of the sacrificial layer, (B) transfer of the film onto a support, and (C) X-ray absorption measurement in a transmission geometry. (b) Reciprocal space L scan around the (1 0 3) reflection from a 20 nm thick STO FS film supported by a Si wafer. The X-ray energy is 12.7 keV. Kiessig fringes are clearly visible in the XRD pattern.

STO [001] films with various thicknesses were epitaxially grown by pulsed laser deposition on STO [001] substrates with an ∼10 nm La_0.7_Sr_0.3_MnO_3_ sacrificial layer in between them. After growth, the heterostructure was immersed in the KI (600 μM) and HCI (50 μM) solution. The chemical solution etched away the sacrificial layer, releasing the STO film into the solution. The released STO film was transferred onto two types of supports, a slightly doped P/B [001] oriented Si-wafer and a Cu-mesh, for characterizations and experiments (see the schematic illustration in Figure 1a). Note that the axis of the STO films and the Si are not correlated, as there is no chemical bonding between them.^26^ The nomenclature of *freestanding* used in this report implies that the films are not epitaxially bounded to the supports. In this work, we have investigated films with 20, 30, 40, and 80 nm thicknesses. The crystal structures of these films were determined using X-ray diffraction (XRD) at the Material Science beamline^27,28^ of the Swiss Light Source. The room temperature XRD pattern from a 20 nm film is shown in Figure 1b. In the spectrum, clear Kiessig fringes can be seen, confirming the film’s high crystallinity with a uniform thickness throughout the probed volume. The refined lattice parameters of a 20 nm freestanding STO film (STO FS) at room temperature are **a** = 3.909(2)°, **b** = 3.906(2)°, **c** = 3.905(4)°, α = 89.99(2)°, β = 90.01(2)°, and γ = 90.00(3)°. These parameters are in good agreement with those from the unstrained cubic structure (bulk **a** = 3.905°, **b** = 3.905°, **c** = 3.905 °, α = 90°, β = 90°, and γ = 90°). Our results suggest the STO FS films have undetectable strain (below 0.1% limited by the measurement precision). The lateral film size is approximately 5 × 5 mm.

## Octahedral Rotations

**Figure 2:**
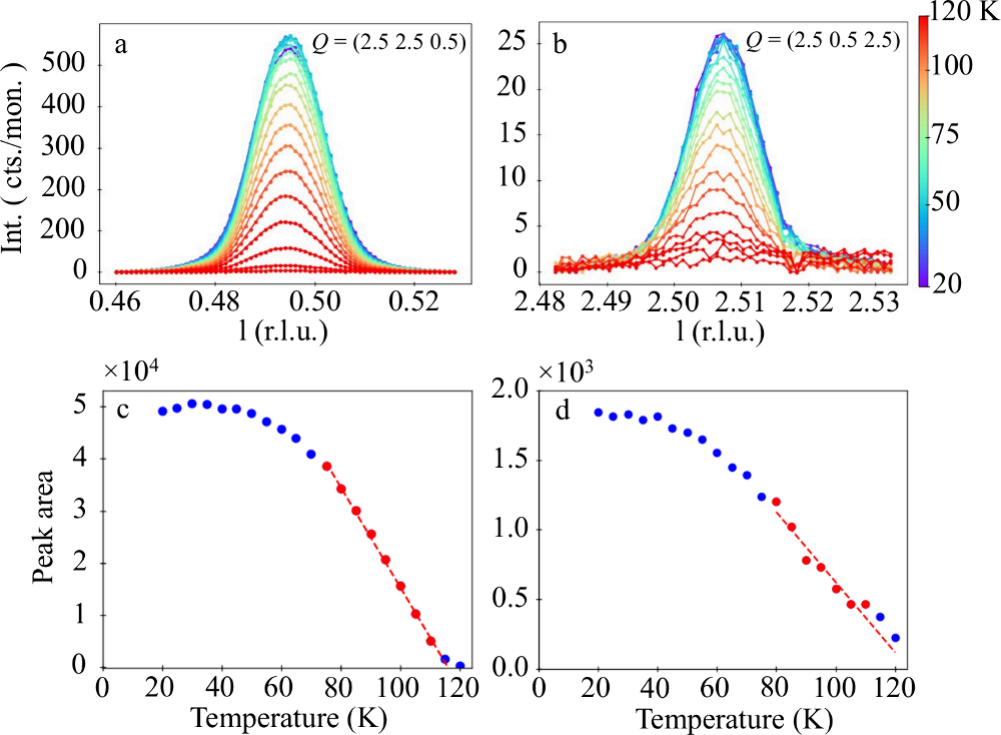
L scans around the superlattice reflections (a) (5/2 5/2 1/2)_c_ and (b) (5/2 1/2 5/2)_c_ as a function of temperature, showing their intensity profiles for the 40 nm thick STO FS film. Fitted peak maximum intensity as a function of temperature for (c) (5/2 5/2 1/2)_c_ and (d) (5/2 1/2 5/2)_c_ reflections. The dotted red line shows the linear fit near the transition that results in the critical temperatures *T*_AFD_ = 116.0 ± 0.5 K and *T*_AFD_ = 123 ± 3 K respectively.

Bulk STO exhibits no TiO_6_ octahedral rotation at high temperature cubic phase; hence, it is denoted as a^0^a^0^a^0^. (Glazer notation:^29^). Below 105 K, the AFD instability arises leading to the tetragonal phase with unit cell parameters *a* = *b* = *a*_0_, *c* = 2 *a*_0_, where *a*_0_ is the lattice constant of the cubic phase. The TiO_6_ octahedra undergo a collective rotation that is out-of-phase between neighbors, hence the rotation is denoted as a^0^a^0^c^–^.^12,30,31^ Because the primitive unit cell is quadrupled, new reflections appear with half-integer indices.^32^ In Figure 2, we show the (5/2 5/2 1/2)_c_ and (5/2 1/2 5/2)_c_ superlattice reflections from a 40 nm FS film (c denotes the pseudocubic notation). The transformation matrix , (45° rotation about the *c*-axis) transforms (5/2 5/2 1/2)_c_ into (5 0 1)_t_ (t denotes the tetragonal notation). This reflection is forbidden in the *I*4/*mcm* space group, because both h and l have to be *even*. Transforming the (5/2 1/2 5/2)_c_ reflection results in (3 2 5)_t_, which is allowed in the *I*4/*mcm* space group. Given the considerable intensity for the (5/2 5/2 1/2)_c_ reflection, the reflection must be of (3 2 5)-type from domains that have octahedra rotated about a different axis. In fact, an in-plane transformation matrix  will correctly transform to (3 2 5)_t_. This implies the coexistence of domains with TiO_6_ octahedra rotated about the in-plane and out-of-plane axes.

This implies the coexistence of domains with TiO_6_ octahedra rotated about the in-plane and out-of-plane axes.

Furthermore, the intensity of these reflections is proportional to the square of the octahedral rotation angle as there is a single free oxygen positional parameter (oxygen at site 8h at (x, x+1/2,0) in the low temperature tetragonal structure that contributes to the structure factor of these reflections. By linearly extrapolating the integrated intensity of these reflections in the temperature range from 75 to 115 K (red dots in Figure 2c,d), we can determine the onset of AFD transition temperature to *T*_AFD_ = 116.0 ± 0.5 K and *T*_AFD_ = 123 ± 3 K for (5/2 5/2 1/2)_c_ and (5/2 1/2 5/2)_c_ reflections, respectively. Despite a slight discrepancy between the *T*_AFD_ values, these temperatures are higher than the bulk value (*T*_AFD_ = 105 K),^16^ suggesting an increased stability of the AFD phase in our STO FS films. First-principles calculations also reported similar results in which the two-dimensional (2D) monolayer STO is found to have a much higher cubic to the tetragonal transition temperature.^33^

We notice that the integrated peak intensities of (5/2 5/2 1/2)_c_ and (5/2 1/2 5/2)_c_ reflections differ by a factor of ∼27 at low temperature (see Figure 2c,d). This large factor cannot be attributed solely to the measurement geometry: the 21° and 4° incidence angles for the (5/2 1/2 5/2)_c_ and (5/2 5/2 1/2)_c_ reflections, respectively, only account for a factor of ∼5 difference in the probing volume. That leaves another factor of ∼5, which we believe is related to the volume ratio between domains with different octahedral rotation axes. Since the (5/2 1/2 5/2)_c_ reflection has a higher intensity, we believe that the AFD domains with an in-plane rotation axis in these very thin FS films dominate. This finding contrasts with results from bulk STO where the octahedral rotation axis aligns with the three principle Cartesian axes without preference. The preferential in-plane rotation axis for AFD is also observed in other STO FS films with varying thickness (see SI).

## Anisotropy of Ti 3*d* States

**Figure 3:**
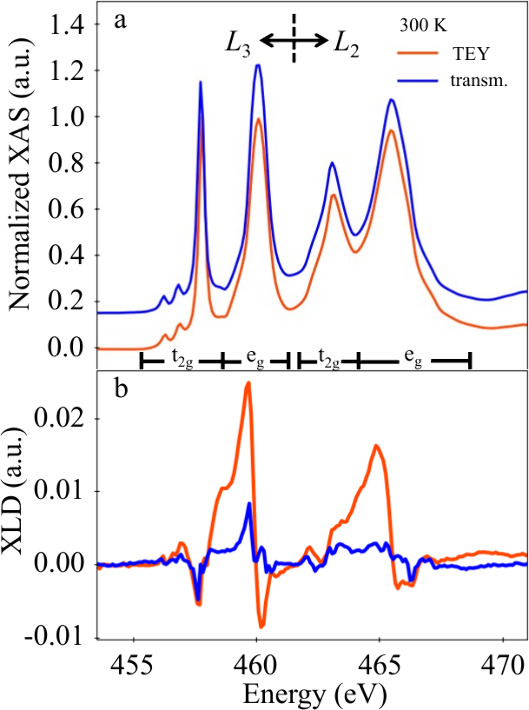
(a) XAS spectra taken at 30° incidence of a 30 nm thick STO FS film at 300 K in transmission (blue) and TEY (orange) modes for linear-horizontal polarization. (b) Average X-ray linear dichroism (*I*_*LV*_ - *I*_*LH*_) of STO FS film with 30 nm thickness supported by a Cu mesh. Data are taken in TEY (orange) and in transmission mode (blue).

Although the AFD structural phase transition does not directly induce the ferroelectricity in bulk STO, a preferential AFD rotation axis, as found in our FS films, may unbalance the domain population of the polarization states, allowing access to anisotropies of the intrinsic electronic structure. To explore this conjecture, we carried out soft X-ray absorption measurements on Cu-mesh-supported STO FS films. Figure 3 shows the X-ray linear dichroism (XLD) of a 30 nm film at the Ti *L*_2,3_-edges. The spectra were recorded in both transmission and total electron yield (TEY) modes at various temperatures between 300 and 40 K.

A small but clear (∼3%) XLD signal is seen in the spectra collected using the surface-sensitive TEY mode (∼3 nm probe depth,^34,35^ orange curve in Figure 3b). This XLD signal is roughly temperature independent (see SI, Figure S2a). On the contrary, the spectra recorded in transmission show a negligible XLD signal (blue curve in Figure 3b). Since the transmission geometry will probe the sample almost homogeneously, it is dominated by the deeper lying layers not probed by the TEY method. Therefore, the difference in the magnitude of the XLD signal between these two detection modes suggests its surface origin. This is plausible because the broken inversion symmetry at the surface and the interface will lead to the splitting of otherwise degenerate *t*_2g_ states, yielding a dichroic contrast.^36,37^ The energy positions of these XLD features permit us to associate them with the unoccupied Ti *d*_*xy*_ (or *d*_*x*^2^-*y*^2^_) states and Ti *d*_*xz/yz*_ (or *d*_3*z*^2^-*r*_) states, respectively.^38^ Their relative sign agrees with the calculations that assume the lower energy for the *d*_*xz/yz*_ states (*d*_*xy*_ > *d*_*xz/yz*_), which is analogous to those in the AlO/SrTiO_3_/NdGaO_3_ interfaces,^39^ The magnitude of the XLD signal, however, is much smaller and is comparable to that of the bare SrTiO_3_^36^ or γ-Al_2_O_3_/SrTiO_3_.^40^

Since we have attributed the observed XLD signal mostly to the surface regions, the Ti t_2g_ orbitals in the inner part of the films remain degenerate. This is somewhat unexpected because of the preferred in-plane rotation axis for AFD that should induce an energetic difference between the *d*_*xy*_ and *d*_*yz*_ orbitals, giving rise to the XLD signal with a slightly different profile. A plausible explanation would be that despite the presence of AFD, the central Ti is little affected, and its orbitals remain mostly degenerate.

## Ferroelectric Soft Mode

The AFD and FE instability are two important ingredients for understanding the phase transition and electronic properties of STO. Since we have observed an enhanced AFD rotation transition temperature compared to that of bulk, it is natural to ask if this behavior could be related to ferroelectricity in these FS films. The concept of a polar soft mode (the lowest optical mode, TO1) is commonly used to explain the dynamics of phase transitions that lead to the ferroelectric state of a material.^11^ When approaching the ferroelectric phase transition temperature *T*_c_ from a high temperature paraelectric state, due to the second-order nature, the frequency of this mode will be lowered (soften) and eventually goes to 0 once the ferroelectric state is established.^41^ However, for oxides, such a transition is close to first order, and the softening is rarely complete. Therefore, one often uses the temperature where the mode is minimal as the onset temperature for ferroelectricity, as in the case of the strained polar STO thin film.

THz time domain spectroscopy (THz TDS) is an ideal technique for detecting the infrared active soft mode. Taking the Fourier transforms of the sample and the reference transmission traces and dividing the resulting spectra yield the spectrum in the frequency domain. For STO, the strongly absorbing nature of this mode makes it detectable in transmission even when the film is extremely thin; however, the presence of growth substrate (such as DyScO_3_) complicates THz TDS to be performed on heterostructures in this geometry. However, for our STO FS films, this is not an issue, and the measurements can be easily performed (see SI for more information). We carried out the THz TDS measurements as a function of temperature and Fourier transformed the results to obtain the THz spectra to determine the mode frequency. Since the THz spectra do not give sufficiently reliable data below ∼0.4 THz, modeling the low frequency response using a Drude–Lorentz model is not trivial. Therefore, the frequency of TO1 mode is extracted based on the minimum in the transmission spectra. Note that the mode is broad, representing a short lifetime, which is typical for low energy soft modes. All THz spectra show the polar transverse optical (TO1) mode; see SI Figure S3 and Figure S4 (for bulk STO, this mode is at ∼2.6 THz at room temperature).

**Figure 4:**
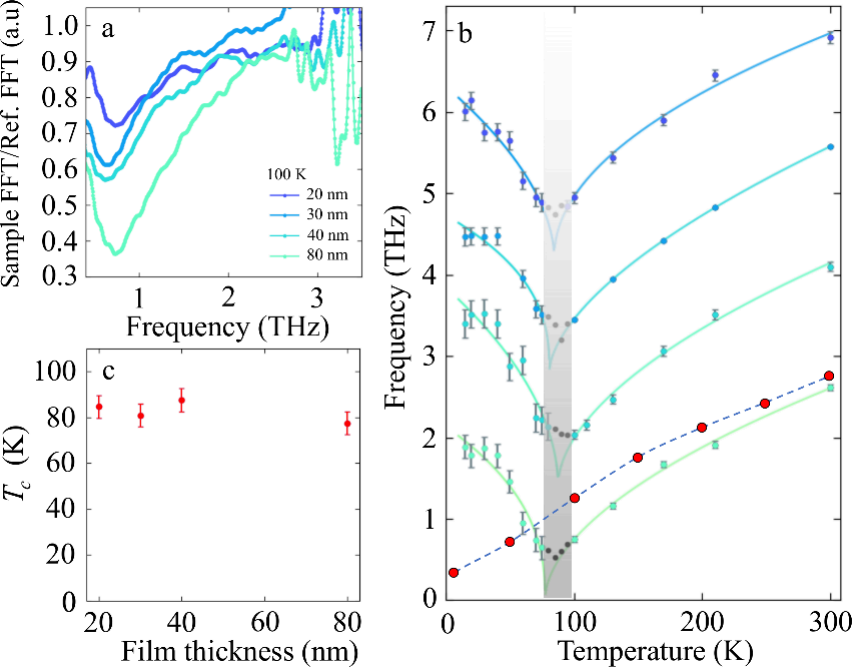
THz TDS as a function of temperature and film thickness. (a) Soft mode at 100 K for all measured film thicknesses. (b) Soft mode frequency as a function of temperature for STO FS film of 20, 30, 40, and 80 nm thickness. The graphs are vertically offset by 1.4 THz, starting from the data set of the 80 nm thick film. The fitting is shown with solid lines, and the points in black were not included in the fit data. The frequency range of inaccessible/imprecise values is highlighted by the shaded area. For comparison, we also included the soft mode behavior of the STO single crystal (red dots), taken from ref (21). (c) Fitted critical temperatures from the disordered and ordered critical exponential fittings as a function of the film thickness. Errors are the standard deviations and obtained from a fit (if visible otherwise smaller as the symbols). For the black points (b), an errors’ estimate is not reliable, and error uncertainities are signficanlty larger.

Figure 4a shows the THz spectra at 100 K for films with different thickness. The spectra are very similar, suggesting only a small variation in the mode frequency between films. When we plot the mode frequency as a function of the temperature in Figure 4b, we see that the mode softens with decreasing temperature and subsequently stiffens below *T*_c_. The behavior is seen for all films with different thickness and is in stark contrast to the bulk (red markers). The minima in Figure 4b correspond to the critical temperatures at which the films become polar, and again, they show little thickness dependence (shaded region). To enable a more precise determination of *T*_c_, we used critical exponent functions (see SI for equations and the fitting results) to fit the data points in ordered (below *T*_c_) and disordered (above *T*_c_) phases (solid lines in Figure 4b). The fitted critical temperature as a function of film thicknesses is plotted in Figure 4(c). The variation is within the precision of our data.

We also looked at the polarization dependence of the TO1 mode. THz spectra of a 30 nm STO FS film with horizontally and vertically polarized THz radiation (both in plane) show no polarization dependence (see SI Figure S5 for details), which indicates randomly distributed in-plane polarized domains. THz transmission with the out-of-plane polarization cannot be assessed due to the limited film thickness. Instead, we used low-temperature piezoresponse force microscopy to measure the out-of-plane ferroelectric polarization and confirmed its absence (see SI for detailed information). Our findings are distinct from those using strained STO films, where the ferroelectricity in those films is polarized in-plane for tensile and out-of-plane for compressive strain, respectively. Since our FS films exhibit negligible residual strain, the presence of these in-plane polarized ferroelectric domains points to other collaborative origins like the AFD domains with the in-plane rotation axis. One could envision that the emergence of AFD-distortion at higher temperature induces charged/polar domain walls in the same direction, modifying the electronic structures around the domain wall regions and promoting the in-plane ferroelectricity at lower temperature.

The lack of a significant thickness dependence of *T*_c_ among our FS films is surprising as one would expect that *T*_c_ should be suppressed when approaching bulk properties. However, it might have some relation to HfO_2_ that gets polar for films below 10 nm^42^ with the difference here that the polarization extents into the near surface region of SrTiO_3_. The most likely explanation is the presence of a small residual stress in the STO FS films, which is within the margin of our error estimated from diffraction measurements. Based on the ferroelectric transition temperature,^11^ the residual strain would be approximately 0.05%. This residual strain could be relevant for applications of freestanding SrTiO_3_ films.

## Discussion

We investigated the canonical oxide perovskite, SrTiO_3_, in its FS form. Structural determination showed that these STO FS films have high crystalline quality and are structurally relaxed to the bulk cubic structure at room temperature. We observed the AFD transition occurs at a slightly higher temperature compared to the bulk, and the AFD shows a preferential in-plane rotation axis. The comparison of XLD spectra recorded using the surface-sensitive TEY mode and bulk-sensitive transmission mode suggests the surface regions are responsible for the observed dichroic contrast. On the other hand, in the inner part of FS films, the Ti *t*_2g_ states remain degenerate, suggesting that octahedral rotation have little impact on the Ti site symmetry. Using THz TDS, we observed the ferroelectric soft mode in all STO FS films. This mode softens with decreasing temperature, causing the films to become polar near 82 K. This behavior is in stark contrast to bulk STO that remains paraelectric down to the lowest temperature. Moreover, the polar domains exhibit in-plane ferroelectricity, and considering the negligible residual strain and AFD with a preferential in-plane rotation axis, our findings might suggest that AFD and ferroelectricity in our FS films are related, either directly or indirectly through e.g. tiny surface strain fields and/or defects. The difference in transition temperature between AFD and FE phase has also been observed in other system. For example, for STO grown on a DyScO_3_ substrate, the AFD transition occurs around 160 K, while the material exhibits polarization at room temperature.^43^

These findings suggest the unique behavior of STO FS films not seen in the bulk and the so-called strain-free STO heterostructure. The contrast showcases the use of crystalline engineering to fine-tune the properties of STO, including the altered AFD transition and emergent ferroelectricity. The ability to manipulate the ground-state properties of STO FS films opens new revenue for inducing material functionality via dimensionality.

## Experimental Section/Methods

### X-ray Diffraction

Structural characterization of all STO FS films were done by X-ray diffraction at the Material Science Beamline at the Swiss Light Source,^25,26^ with an X-ray energy *E* = 12.7 keV and 500 × 500 μm beam size. Lattice parameters at room temperature were thoroughly benchmarked with a series of measurements of structural Bragg peaks for the 20 nm thick STO FS films transferred on a Si-wafer piece. Various combinations of peak positions were used to refine the lattice parameters, which are found to be cubic at room temperature.

### Temperature-Dependent X-ray Diffraction

XRD measurements of the superlattice reflections of STO FS films [001] of 20 and 40 nm thicknesses were performed at the i16 beamline at Diamond light source.^44,45^ The X-ray energy was set to 11 keV with a beam size of 180 μm x 30 μm, and temperatures ranged from 120 to 20 K.

### X-ray Linear Dichroism

X-ray absorption spectra of a 30 nm thick STO FS film were measured as a function of temperature in both transmission and TEY modes at the X-TREME beamline^38^ at the Swiss Light Source. The measurements were performed with 30° grazing incidence X-rays with linear-horizontal and linear-vertical polarization and a (250 μm × 30 μm) beam size probing mainly out-of-plane and in-plane directions, respectively. XLD was corrected for tiny energy shifts between the spectra in different X-ray polarizations at normal incidence. Energy-dependent data in both polarizations was acquired and normalized by the average of the XAS maxima (XAS calculated as the average of both polarizations). XLD for each temperature consists of *I*_LV_ – *I*_*LH*_.

### THz Time Domain Spectroscopy

THz-TDS experiments were performed at the Institute of Quantum Electronics at the ETH. In the TDS measurement, the STO FS films were transferred onto a Si wafer, because Si has a good transparency in the THz frequency range. Transmission through the sample was measured in the time domain and compared to that through a Si wafer reference. We used a standard THz-TDS setup that allows the measurement of the transmission in the frequency range of ∼0.5–3.0 THz.^44^ The setup consists of a 250 kHz repetition rate laser system that emits sub 40 fs long pulses of 800 nm wavelength photons, with an average output power of about 1.5 W, and the THz pulses are generated in a spintronic emitter and detected using electro-optic sampling in a 1 mm thick ZnTe crystal.

### Piezoresponse Force Microscopy

*Piezoresponse force microscopy* hysteresis measurements were acquired using a commercial cryogenic scanning probe microscope system (attoAFM I, Attocube) with a closed-cycle cryostat (attoDRY 2100 with 9 T magnet, Attocube) at 1.6 and 70K. The switching spectroscopic technique was carried out under contact-resonance mode with the commercial platinum silicide (PtSi) coated tips with a spring constant of 2.8 N/m (NANOSENSORS PtSi-FM). The tip was driven with an AC voltage amplitude of about 2 V and was working at a contact-resonance frequency of about 300 kHz. The off-field hysteresis loops were obtained via the switching spectroscopic technique with an arbitrary waveform generator (G5100A, Picotest).

## Supporting Information Available

X-ray diffraction conditions and additional data, temperature dependence of XLD, further experimental info on THz time domain spectroscopy, Low-temperature piezoresponse force microscopy (LT_PFM) and fitting process. Supporting Information is available from the Wiley Online Library. Experimental data will be available from SciCat system under discovery.psi.ch with doi: http://doi.psi.ch/detail/10.16907%2F24dd17b1-b637-4e69-a38a-3b7bbfb3e4e8 (PDF)
