# Supplementary material for: Antiferrodistortive and Ferroeletric Phase Transitions in Freestanding Films of SrTiO3
Source: Nano Lett. 2025 May 1;25(19):7651–7. doi: 10.1021/acs.nanolett.4c05664 (PMC12082687; doi:10.1021/acs.nanolett.4c05664)
Supplement: Supplementary file 1 — nl4c05664_si_001.pdf [file nl4c05664_si_001.pdf]

Supporting Information

Antiferrodistortive and ferroeletric phase transitions in freestanding films of $SrTiO_3$

Ludmila Leroy[1]†, Shih-Wen Huang[1]†, Chun-Chien Chiu[2], Sheng-Zhu Ho[2], Janine Dössegger[3], Cinthia Piamonteze[1], Yi-Chun Chen[2], Elsa Abreu[3], Alessandro Bombardi[4], Jan-Chi Yang[2] and Urs Staub[1]**

[1]PSI Center for Photon Science, Paul Scherrer Institute, Forschungsstrasse 111, 5232 Villigen, Switzerland
[2]Department of Physics, National Cheng Kung University, Tainan 701, Taiwan
Center for Quantum Frontiers of Research & Technology (QFort), National Cheng Kung University, Tainan, 701401 Taiwan
[3]Institute for Quantum Electronics, ETH Zürich, Auguste-Piccard-Hof 1, 8093 Zürich, Switzerland
[4]Diamond Light Source Ltd, Diamond House, Harwell Science & Innovation Campus, Didcot, Oxfordshire, OX11 0DE

Incident angles and octahedral rations

X-ray diffraction (XRD) was performed in the vertical scattering geometry, with the (0 0 1) plane aligned horizontally in the laboratory frame. At 11 keV, the θ and 2θ angles for the $(5/2\ 5/2\ 1/2)_c$ and $(5/2\ 1/2\ 5/2)_c$ diffraction peaks are 31.02° and 62.04°, respectively. The angle between $(5/2\ 5/2\ 1/2)_c$ and (0 0 1) is 81.9°. Thus, the incident angle for this diffraction peak is approximately 4.36° (31.02°*sin(90°-81.9°)). Similarly, the angle between $(5/2\ 1/2\ 5/2)_c$ and (0 0 1) is 45.56°, leading to an incident angle of approximately 21.72° (calculated as 31.02°*sin(90°-45.56°)). In Figure S1 (a) and S1(b) we show the superlattice reflections $(5/2\ 5/2\ 1/2)_c$ and $(5/2\ 1/2\ 5/2)_c$ as a function of temperature of 20 nm STO FS on Si wafer. We fitted the spectra with a Gaussian function on top of a linear background, and the area under the Gaussian function was used as the superlattice reflection peak area. By linearly extrapolating the peak area in the temperature between 80 K and 110 K to 0 K, the obtained T_{AFD} is approximately 117 K and 121 K for the $(5/2\ 5/2\ 1/2)_c$ and $(5/2\ 1/2\ 5/2)_c$ reflections, respectively. The intensity difference between these two reflections, though smaller than the difference observed in the 40nm films, confirms again a preferential in-plane octahedral rotation axis. Notably, as these two superlattice reflections originate from two different orientations of domains, the statistically significant difference between their T_{AFD} also indicates that they not only are unevenly populated, but also have slightly different energetics.

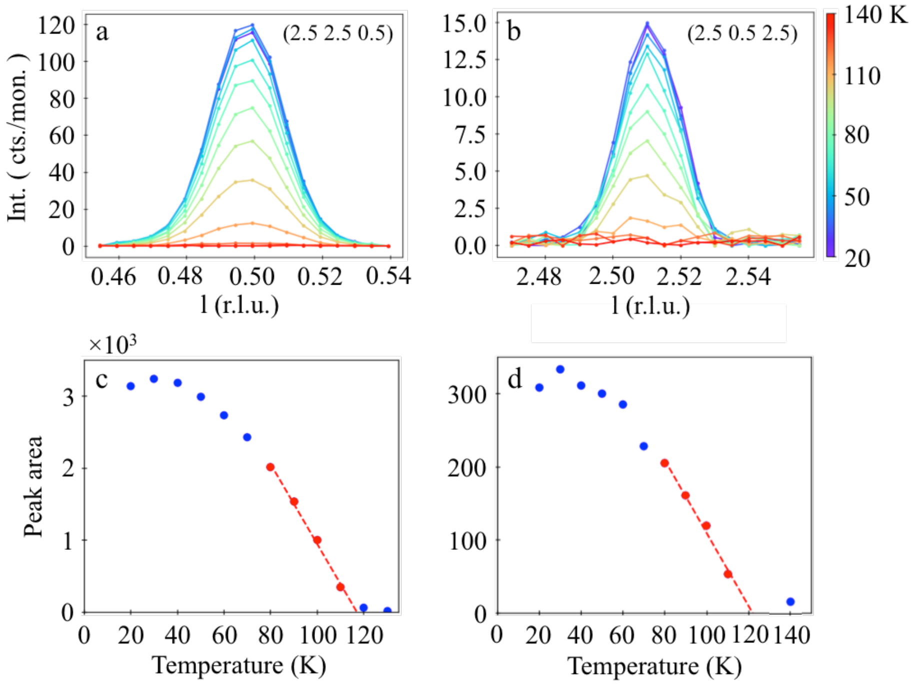

Figure S1: (a) $(5/2\ 5/2\ 1/2)_c$ and (b) $(5/2\ 1/2\ 5/2)_c$ superlattice reflections as a function of temperature of a 20 nm STO FS film transferred onto a Si wafer. The corresponding peak area from a Gaussian fitting with a linear background are shown in panels (c) and (d), respectively. Linear extrapolation (red dashed line) of data points from the temperature range 80 K and 110 K (red markers) results in the AFD transition temperature (T_{AFD}) of approximately 117 K and 121 K for $(5/2\ 5/2\ 1/2)_c$ and $(5/2\ 1/2\ 5/2)_c$, respectively.

Temperature-dependent XLD

Figure S2 (a) and (b) show the temperature-dependent Ti $L_{2,3}$-edge X-ray linear dichroism (XLD) of a 30 nm STO-FS film measured in the total electron yield (TEY) mode and the transmission mode, respectively. To compare the XLD measured with different mode, the XAS spectra were normalized using the spectral weight at the L_3 t_{2g}

sector (energy from 455 eV to 458.5 eV representing the unoccupied Ti 3d t_{2g} orbitals, see also Fig. 3a).

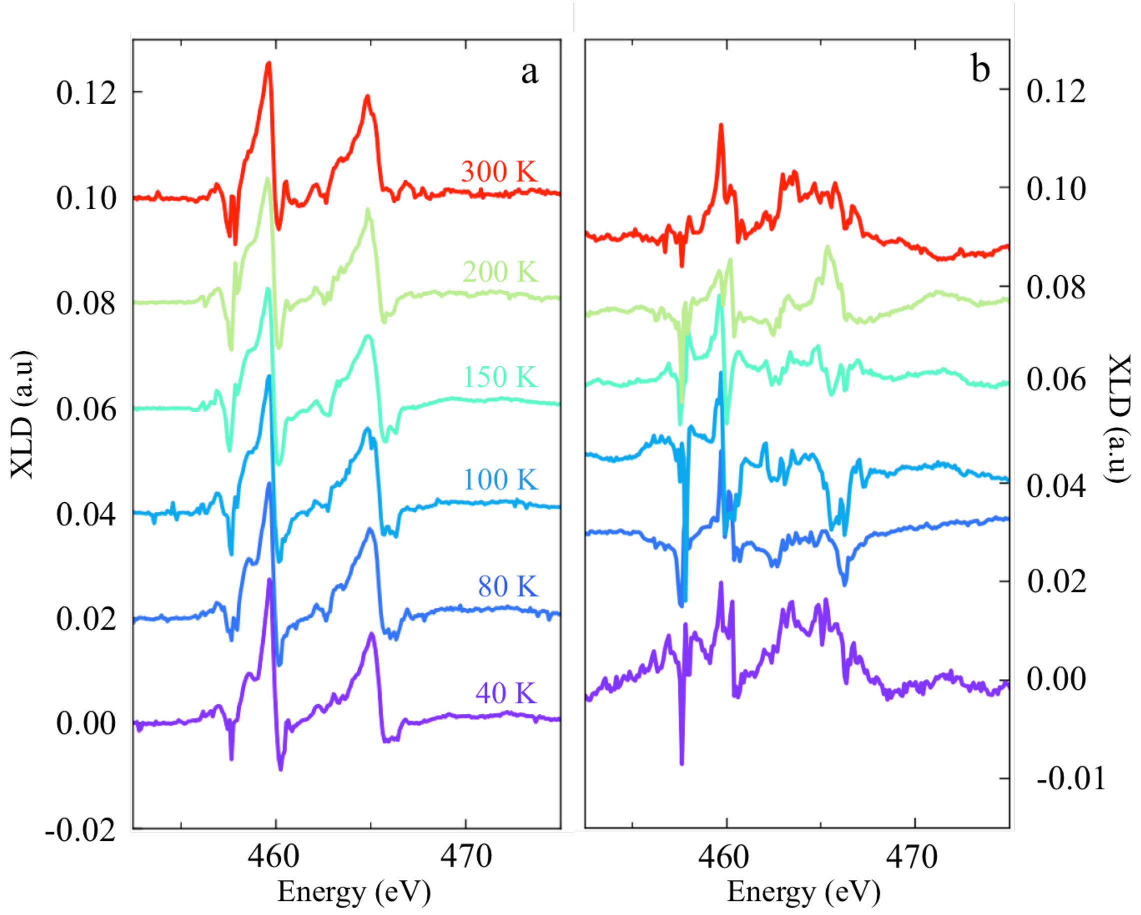

Figure S2: Temperature-dependent X-ray linear dichroism at Ti $L_{2,3}$ – edge of a 30 nm STO-FS film collected in (a) total electron yield (measuring the sample-to-ground drain current) and (b) transmission (X-ray transmission) modes.

Ferroelectric soft mode demonstrated by THz time domain spectroscopy (TDS)

Figure S3 (a) shows the THz transmission of a 30 nm STO-FS film transferred onto (001) Si wafer (color lines) and the reference (bare Si wafer, black dash line) as a function of time delay at selected temperatures. In the time-domain spectra, we can see a small

amplitude difference between the STO-FS and the reference, indicating a slight change in transmission properties with temperature. The corresponding frequency domain spectra were obtained by Fourier transform the time-domain signal of STO-FS film on Si and divided it by that of the reference (see Fig. S4). The frequency of the TO1 mode is defined by the minimum between 0.4 and 2.5 THz.

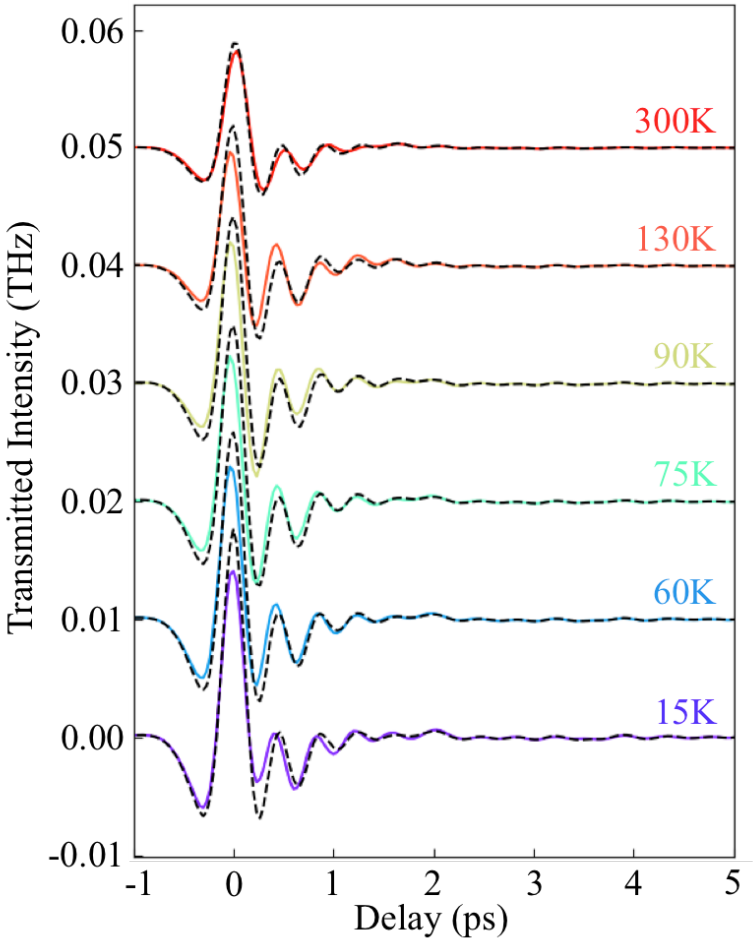

Figure S3: THz time domain spectra of a 40 nm thick $SrTiO_3$ freestanding film (STO FS) on a Si wafer (color lines) and the Si wafer reference (black dash lines) at different temperatures.

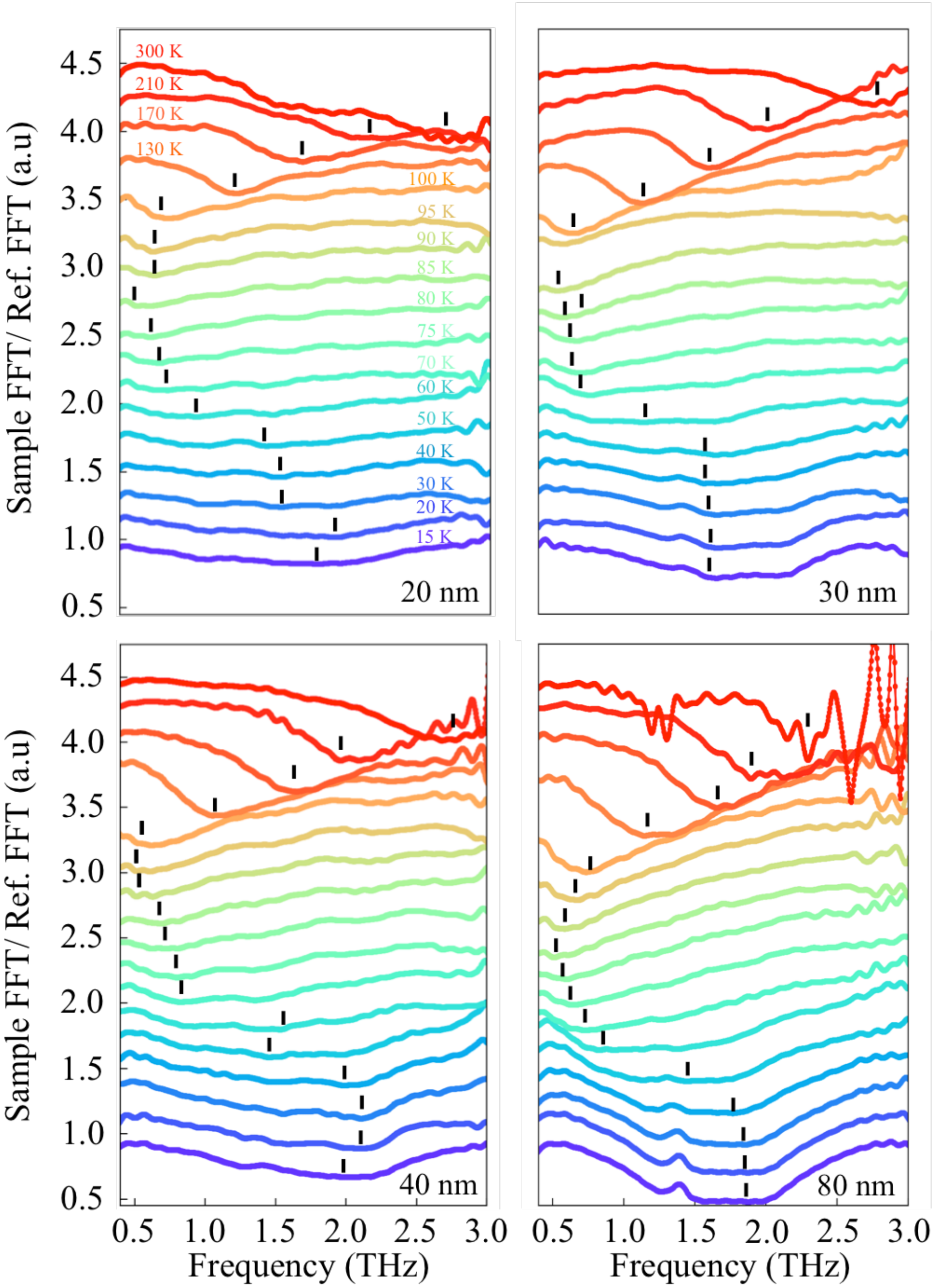

Figure S4: Temperature-dependent THz frequency domain spectra of STO-FS films with different thickness. The frequency of TO1 mode is denoted by a black vertical tick.

Equations E1: Fit functions E1a (for T>T_C) and E1b (for T<T_C) for extracting the minimum of the soft mode (TO1) frequency in Figure 4(b). This minimum is the ferroelectric transition temperature T_C.

$$F(T) = a_1 \cdot ((T - T_c)/T_c)^{b_1} \qquad \mathrm{E}\mathbf{1a}$$
$$F(T) = a_2 \cdot (-(T - T_c)/T_c)^{b_2} \qquad \mathrm{E}\mathbf{1b}$$

The fitted results are list in table T1.

	Disodered (T>T_C)			Ordered (T<T_C)		
	a1	b1	T_c	a2	b2	T_c
20 nm	1.73±0.16	0.50±0.07	85±5	2.1±0.2	0.55±0.23	85±5
30 nm	1.51±0.03	0.60±0.01	81±5	1.9±0.2	0.31±0.1	81±5
40 nm	1.67±0.16	0.55±0.07	87±5	2.4±0.2	0.52±0.23	87±5
80 nm	1.44±0.2	0.55±0.06	77±5	2.1±0.2	0.41±0.13	77±5

Table T1: Parameters from fitting the data points in Figure 4(b) with fit function E1a (for $T > T_C$) and E1b (for $T < T_C$). The extracted temperature is assigned to the ferroelectric transition temperature at which the phonon soft mode frequency shows a minimum.

To validate the in-plane domain orientation, we perform the TDS with both linear horizontal and linear vertical polarizations on a 30nm STO-FS film. The results are shown in Figure S5. Although both polarization modes are in the TiO_2 plane, the contrast may indicate possible preferential in-plane orientation. We can see that both polarizations results in similar ferroelectric transition temperature of approximately 80 K, indicating that the ferroelectric domains do not have preferred orientations.

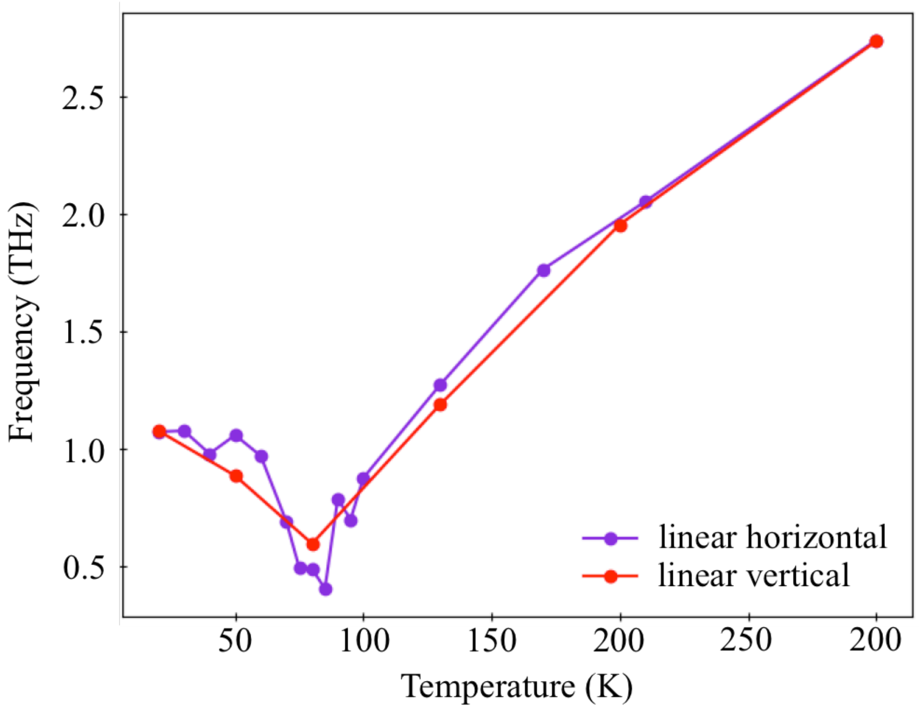

Figure S5: Extracted soft mode frequency of a 30 nm STO-FS film on Si wafer from TDS measurements using linear vertical and horizontal THz E-field polarizations.

Low-temperature piezoresponse force microscopy (LT_PFM)

To further exam the ferroelectric behavior of STO-FS below the Curie temperature, we also employed low-temperature piezoresponse force microscopy (LT_PFM) to assess the ferroelectric switching properties. The switching spectroscopic technique was also utilized

to minimize the artifacts from the electrostatic interaction between the tip and sample during the hysteresis measurement. Figure S6a and S6b show the out-of-plane piezoresponse force microscopy signals as a function of sample bias from -30 V to 30 V. Both out-of-plane amplitude and phase spectra exhibit the absence of a nonlinear polarization switching behavior because they do not show the 180 degrees correlation between amplitude and phase. The absence of such correlation suggests the absence of out-of-plane piezoresponse.

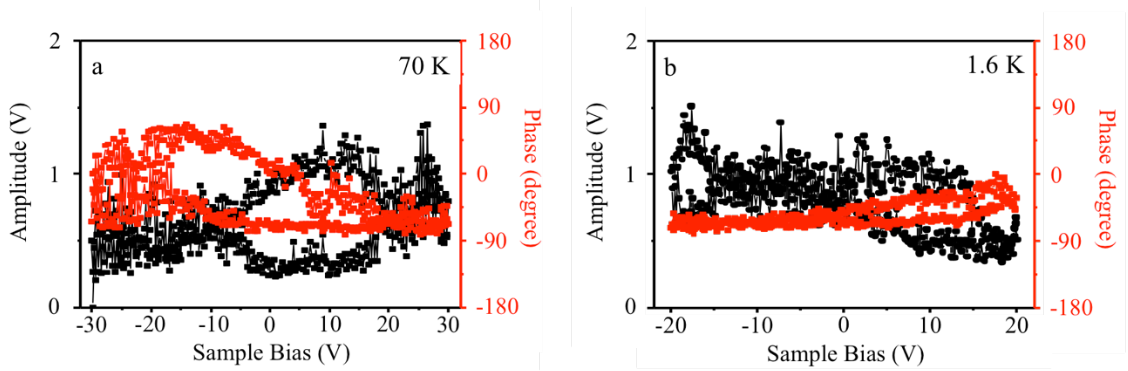

Figure S6: Piezoresponse force microscopy characterizations at (a) 70 K and (b) 1.6 K
